# Editorial for the Special Issue on Advances in Micro and Nano Manufacturing: Process Modeling and Applications

**DOI:** 10.3390/mi12080970

**Published:** 2021-08-16

**Authors:** Davide Masato, Giovanni Lucchetta

**Affiliations:** 1 Department of Plastics Engineering, University of Massachusetts Lowell, 1 University Avenue, Lowell, MA 01854, USA; 2 Department of Industrial Engineering, University of Padova, Via Venezia 1, 35131 Padova, Italy

Micro- and nano-manufacturing technologies have been developed in research and industrial environments to support product miniaturization and the integration of new functionalities. The technological development of new materials and processing methods has been supported by developing predictive models, which can simulate the interactions between materials, process states, and product properties. Compared with the conventional manufacturing scale, micro- and nano-scale technologies require different mechanical, thermal, and fluid dynamics phenomena to be studied and modeled. To fully realize the micro- and nano-scale potential, several challenges still need to be overcome. Among them are understanding the material processing behavior at a reduced scale, developing design criteria, validating process control and monitoring strategies, and defining quality control procedures. 

This Special Issue showcases 12 state-of-the-art examples of modeling and simulation in micro- and nano-manufacturing processes. The papers that are published in this Special Issue explore the following micro- and nano-manufacturing processes: laser texturing [1,2], 3D printing [3,4], grinding [5,6], turning [7,8], and electrochemical machining [9,10]. The remaining papers cover lithography-based manufacturing [11], and micro- and nano-scale design aspects [12].

Laser texturing technologies exploit electromagnetic energy, to melt and vaporize the material on the surface, creating a texture. Ultrafast texturing is a recent development in laser technologies, which has many peculiarities and allows the machining of different textures. Ultrafast lasers are pulsed lasers that are characterized by a duration of a few nanoseconds, and a frequency ranging between one and several hundreds of kHz. Ultrafast laser systems are characterized by low-power characteristics (i.e., units of Watts); however, the use of high-power light pulses allows for high values of energy to be focused on the workpiece surface. Kažukauskas et al. used a femtosecond laser to polish the surface of a transparent biocompatible polymer for body implants [1]. The results showed that the polishing process depends on the thermal heat flux, which initiates thermos-dynamical phenomena that change the surface roughness of the sample. Piccolo et al. used a femtosecond laser to create a submicron texture on a metallic mold, which was then replicated by microinjection molding [2]. The texture replication resulted in the functionalization of a plastic surface, which showed different static wetting behavior, as a function of the texture replication attained with the molding process.

Recent innovations in 3D printing technologies allow the direct manufacturing of micro- and nano-scale products, such as microfluidics, and the generation of fine details on manufacturing tools. Dempsey et al. used a digital light processing (DLP)-based inverted stereolithography process, to produce thermoset polymer-based tooling for micro injection molding [3]. The results show that the resistance of the soft tool is related to its dynamic temperature distribution during the process, and its ability to dissipate the heat that is convected by the hot polymer melt. Chen et al. developed a sample library of standardized components and connectors, manufactured using a multi-jet modeling technology, using a print head that jets the photopolymer and the waxy support material [4]. Their modular microfluidic system offers potential for realizing mass-production complexes and multiplex systems for the commercialization of microfluidic platforms.

Grinding, at the nano-scale level, can be efficiently employed to create surfaces with ultrahigh precision, by removing a few atomic layers from the substrate. Manea et al. developed an arithmetical model to calculate the nano-scale grinding force required to machine optical glass, and analyzed how processing parameters can be controlled to achieve high surface and subsurface quality [5]. Karkalos et al. focused on molecular dynamics modeling with multiple abrasive grains, to study the effect of spacing between the adjacent rows of abrasive grains and the effect of the rake angle of the abrasive grains [6]. They evaluated the grinding forces and temperatures, ground surface quality, chip formation, and subsurface damage of the substrate.

Skrzyniarz studied the minimum chip thickness formation during the longitudinal turning of steel [7]. The analysis of the machined workpiece showed that the depth of cut and the feed rate affects the minimum chip thickness. Elastic-plastic deformation and ploughing were observed when the feed rate was lower than the cutting edge radius. You et al. carried out an experimental investigation, focusing on laser-assisted turning by the in-process heating of tungsten carbide, for precision glass molding [8]. The process guides the laser beam passes through the transparent tool, to heat the material locally, thereby decreasing the ultra-high tungsten carbide hardness and altering the fracture toughness as soon as the tool interacts with the material. Through an experimental study, the technology was shown to improve machinability, making the ductile turning of hard and brittle materials possible, and has been confirmed as a viable solution for hard and brittle material ultra-precision machining.

Electrochemical machining uses energy (i.e., chemical, electrical, thermal) to remove material from the workpiece’s surface, without physical contact. He et al. studied radial ultrasonic rolling electrochemical micromachining, which is a process that is characterized by the feeding of small and rotating electrodes, aided by ultrasonic rolling, to create an array of pits [9]. Fan et al. analyzed a method of electrochemical machining, using a conductive masked porous cathode and jet electrolyte supply, to generate micro-grooves with high machining localization [10].

A lithography process involves transferring a geometric pattern from a mask to a photoresist-coated semiconductor wafer surface. Geng et al. used a high-precision lithography simulation model for thick SU-8 photoresist, based on the waveguide method, to calculate light intensity in the photoresist and predict the profiles of developed SU-8 structures [11]. 

Langford et al. developed a methodology to calculate the forces acting upon the intraosseous transcutaneous amputation prosthesis, which was designed for use in a quarter amputated femur [12]. The design approach focused on a failure feature, shaped similarly to a safety notch, which would stop excessive stress permeating the bone, causing damage to the user. The topology analysis identified critical materials and local maximum stresses when modeling the applied loads.

The guest editors would like to take this opportunity to thank all the authors for submitting their papers to this Special Issue, and for contributing to the diffusion and development of micro- and nano-manufacturing technologies. We would also like to thank all the reviewers for dedicating their time and helping to improve the quality of the submitted papers.
